# Direct comparison of oligochaete erythrocruorins as potential blood substitutes

**DOI:** 10.1002/btm2.10067

**Published:** 2017-07-19

**Authors:** Devon Zimmerman, Matthew DiIusto, Jack Dienes, Osheiza Abdulmalik, Jacob J. Elmer

**Affiliations:** ^1^ Dept. of Chemical Engineering Villanova University 800 East Lancaster Avenue Villanova PA 19085; ^2^ Div. of Hematology, Abramson Building The Children's Hospital of Philadelphia 34th St. & Civic Center Blvd Philadelphia PA 19104

**Keywords:** blood substitute, erythrocruorin, hemoglobin, hemoglobin‐based oxygen carrier, oxygen transport, protein stability, size exclusion chromatography

## Abstract

While many blood substitutes are based on mammalian hemoglobins (e.g., human hemoglobin, HbA), the naturally extracellular hemoglobins of invertebrates (a.k.a. erythrocruorins, Ecs) are intriguing alternative oxygen carriers. Specifically, the erythrocruorin of *Lumbricus terrestris* has been shown to effectively deliver oxygen in mice and rats without the negative side effects observed with HbA. In this study, the properties of six oligochaete Ecs (*Lumbricus terrestris*, *Eisenia hortensis*, *Eisenia fetida*, *Eisenia veneta*, *Eudrilus eugeniae*, and *Amynthas gracilis*) were compared in vitro to identify the most promising blood substitute candidate(s). Several metrics were used to compare the Ecs, including their oxidation rates, dissociation at physiological pH, thermal stability, and oxygen transport characteristics. Overall, the Ecs of *Lumbricus terrestris* (LtEc) and *Eisenia fetida* (EfEc) were identified as promising candidates, since they demonstrated high thermal and oligomeric stability, while also exhibiting relatively low oxidation rates. Interestingly, the O_2_ affinity of LtEc (*P*_50_ = 26.25 mmHg at 37 **°**C) was also observed to be uniquely lower than EfEc and all of the other Ecs (*P*_50_ = 9.29–13.62 mmHg). Subsequent alignment of the primary sequences of LtEc and EfEc revealed several significant amino acid substitutions within the D subunit interfaces that may be responsible for this significant change in O_2_ affinity. Nonetheless, these results show that LtEc and EfEc are promising potential blood substitutes that are resistant to oxidation and denaturation, but additional experiments will need to be conducted to determine their safety, efficacy, and the effects of their disparate oxygen affinities in vivo.

## 1 INTRODUCTION

Donated blood is the safest and most effective way to treat patients in hemorrhagic shock. However, donated blood must also be constantly refrigerated and expires 42 days after donation.^1^ Consequently, donated blood is typically unavailable in remote locations that lack proper storage facilities. These limitations have motivated the development of alternative oxygen carrying solutions that do not require refrigeration, have a long shelf life, and are as safe and effective as donated blood.

The majority of blood substitutes are hemoglobin‐based oxygen carriers (HBOCs) that utilize human (HbA) or bovine (bHb) hemoglobin.^2^ Examples of HBOCs include Hemopure® (polymerized bHb made by OPK Biotech),^3^ Hemospan® (PEGylated HbA made by Sangart),^4^ PolyHb‐SOD‐CAT (hemoglobin cross‐linked via glutaraldehyde with superoxide dismutase and catalase),^5^ and many others.^6^, ^7^, ^8^ Unfortunately, despite early promise, most HBOCs eventually failed Phase III clinical trials due to adverse reactions such as vasoconstriction, hypertension, and stroke. These effects have been attributed to nitric oxide scavenging ( $Hb\left[{Fe}^{2+}\right]O_{2}+NO\to Hb[{Fe}^{3+}]{NO}_{3}^{-}$) and oxidation of the heme iron ( $Hb\left[{Fe}^{2+}\right]O_{2}+O_{2}^{-}\to Hb\left[{Fe}^{3+}\right]+\;O_{2}^{-}$).^2^, ^9^, ^10^ New HBOC products are being developed to solve these problems, including OxyVita (a higher MW “zero‐link” polymerized bHb),^11^, ^12^, ^13^, ^14^ HemoTech (an anti‐inflammatory ATP cross‐linked bHb),^15^ and pPolyHb (a polymerized porcine Hb), but clinical data are not yet available for these products.^16^, ^17^

The limited success of intracellular mammalian Hbs has also motivated researchers to investigate the naturally extracellular hemoglobins of invertebrates (aka Erythrocruorins or Ecs). Ecs from a variety of organisms have been studied, including annelids,^18^ mollusks,^19^ insects,^20^, ^21^ snails,^22^, ^23^ and many more.^24^ Overall, the most thoroughly investigated Ecs are from the annelids *Lumbricus terrestris* and *Arenicola marina,* which are both huge macromolecular complexes (MW ∼3.6 MDa). For example, the structure of LtEc (obtained via X‐ray crystallography) consists of 144 globins and 36 linker subunits.^25^ LtEc assembly begins when 4 globins form a tetramer, which can then associate with other tetramers to form a dodecamer (∼208 kDa). The linker chains bind the dodecamers to form a protomer that associates with 11 other protomers to yield a hexagonal bilayer (HBL) of protomers that is ∼30 nm across.

The Ec of the marine polychaete *A. marina* (AmEc, also known as Hemarina®) has been successfully utilized for oxygen preservation in tissue culture,^26^ oxygen transfer in bioreactors,^27^ and organ preservation.^28^ AmEc has also been successfully transfused into mice and hamsters without eliciting an immune response or significant changes in blood pressure.^29^, ^30^ However, AmEc was observed to quickly dissociate from the HBL into dodecamers when exposed to the relatively low ionic strength of human plasma in in vitro studies conducted at 37°C, pH 7.4.^29^ In contrast, the Ec of the terrestrial oligochaete *L. terrestris* (LtEc) does not dissociate in human plasma.^31^ However, transfusions of both AmEc and LtEc effectively maintain oxygen delivery in mice, rats, and hamsters without eliciting an immune response or increase in blood pressure.^31^, ^32^, ^33^ Altogether, these promising results indicate that both AmEc and LtEc could be safe and effective blood substitutes.^25^

In addition to AmEc and LtEc, several other Ecs with unique properties have also been described.^24^, ^34^ For example, the oxygen affinity of some Ecs can be relatively high, including the polychaete *Branchipolynoe symmytilida* (*P*_50_ = 0.9–1.4 mmHg at 20°C, pH 7.5)^35^ and the giant (∼3 m in length^36^) Gippsland worm (*P*_50_ = 2 mmHg at 25°C, pH 7.5).^37^ In contrast, the “chlorocruorins” of *Eudistylia vancouverii* and *Potamilla leptochaeta* have a modified heme group that gives them a green appearance and significantly decreases their oxygen affinity (*P*_50_ = 145 mmHg and 155 mmHg, respectively, at 20°C, pH 7.4).^38^, ^39^ Meanwhile, the Ecs from *Riftia pachyptila* and *Oligobranchia mashikoi* posess a unique spherical structure that consists solely of globin subunits (MW ∼400 kDa).^40^, ^41^ Annelid Ecs have also been discovered in extreme environments. For example, the marine worm *Alvinella pompejana* is a hydrothermal vent dwelling marine worm that thrives in an environment that is anoxic, rich in CO_2_ and sulfide, and can tolerate temperatures that vary from 2 to 350°C.^42^, ^43^

While the unique properties of these exotic Ecs may be attractive, many of these species are prohibitively rare or difficult to obtain. In contrast, terrestrial oligochaete worms are available in relatively large quantities and low costs due to their prevalence in the bait and composting industries. Many of these terrestrial worms have been studied individually (e.g., LtEc^25^ and *Glossoscolex paulistus* (GpEc)^44^, ^45^, ^46^, ^47^, ^48^), but very few direct comparisons of their properties are available.^34^, ^45^ The purpose of this study is to directly compare the biophysical properties of six erythrocruorins from commercially available oligochaetes, including *Lumbricus terrestris* (LtEc, Canadian nightcrawler), *Eisenia hortensis* (EhEc, European nightcrawler),^49^ *Eisenia fetida* (EfEc, red wiggler),^50^ *Eisenia veneta* (EvEc, panfish worm),^51^ *Eudrilus eugeniae* (EeEc, African nightcrawler),^52^ and *Amynthas gracilis* (AgEc, Alabama jumper).^53^, ^54^ Specifically, the structural/thermal stability, O_2_ affinity, and oxidation rates of each Ec were compared to identify the most stable Ec(s). These oligochaetes are derived from three different families (*Lumbricidae*, *Eudrilidae*, *and Megascolecidae*) and their detailed phylogenetic relationships are shown in Supporting Information Figure 1.

## 2 RESULTS

### 2.1 Ec purification

Most of the Ecs were easily purified on 500 kDa MWCO tangential flow filter (TFF) cartridges, due to the high MW of the HBL (∼3.6 MDa). In contrast, only a small amount of red AgEc was retained by the 500 kDa filter, while a much larger volume of dark brown filtrate was observed. This observation suggests that the AgEc may have rapidly dissociated during purification, possibly due to the oxidation of the heme iron, thereby allowing it to permeate the 500 kDa filter. Nonetheless, the relatively small amount of red retentate sample obtained for AgEc was used for subsequent experiments. The oxidation level of the purified AgEc sample was relatively high (24.7%, see Table 1) but the oxidation levels of the other purified Ecs (shown in Table 1) were all relatively lower (4–15% Fe^3+^).

**Table 1:** Oxidation level (%Fe^3+^) of Ecs after TFF purification

	LtEc	EfEc	EhEc	EvEc	EeEc	AgEc
Oxidation level (% Fe^3+^)	4.0%	14.5%	7.8%	7.4%	6.2%	24.7%

### 2.2 PAGE analysis

**Figure 1:**
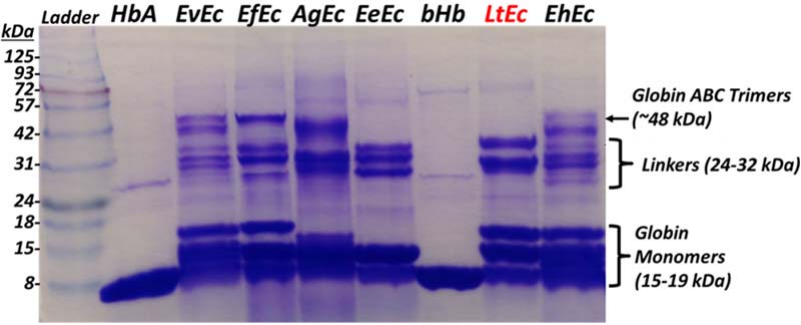
PAGE analysis of TFF‐purified Ecs. All samples were diluted to an absorbance at 540 nm of *A* = 0.1 with 20 mM Tris (pH 7.0), and ∼1.76 μg of each Ec and ∼1.12 μg of each Hb was loaded into each lane and run at 35 V for 10 min, followed by 125 V for ∼2 hr. Protein bands were then stained with Coomassie Blue

Following TFF purification, all Ecs were analyzed on a 10% acrylamide/glycine gel (see Figure 1). HbA and bHb were also included as MW standards that contain only globin subunits (16 kDa; no linker subunits). Overall, each Ec appeared to be highly pure, showing only the characteristic Ec band pattern with multiple globin monomers around 15–18 kDa and multiple linker subunits ranging from 24 to 32 kDa. However, while EvEc, EfEc, LtEc, and EhEc all displayed at least three distinct globin bands, AgEc and EeEc only appeared to have two distinct globin bands. This observation suggests that the MW of one of the AgEc and EeEc globins may be significantly different than the other Ecs. It is worth noting that the A subunit of LtEc was reported to be glycosylated with glycans that are 1.4–1.9 kDa.^55^ Therefore, this difference in band patterns may reflect a difference in glycosylation of one of the subunits, but without primary sequence data for AgEc and EeEc it is unclear why they lack a higher MW globin band around 18 kDa. Nonetheless, the band patterns of the linker subunits of each Ec were highly similar. EvEc, EfEc, AgEc, and EhEc did display higher MW bands, but these may be attributed to unreduced disulfide‐linked ABC trimers (MW ∼48 kDa), which are a common structural feature of Ecs.^56^ Future experiments with electron spray ionization mass spectrometry will need to be conducted to determine the exact MW of each Ec and their glycosylation patterns.

### 2.3 Size exclusion chromatography: Structural stability

**Figure 2:**
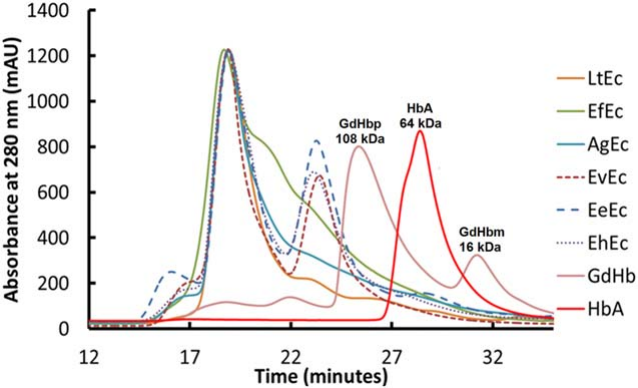
SEC elution profiles of Ecs. All Ecs were analyzed with a BioRad Enrich SEC 650 10 x 300 column using 20 mM Tris, pH 7.4 at a flow rate of 0.5 ml/min. HbA (MW HbA = 64 kDa) and GdHb (MW GdHb monomer = 16 kDa) are shown as a MW standards

The SEC elution profiles of each purified Ec are shown in Figure 2 (mobile phase = 20 mM Tris, pH 7.4). The elution profile of HbA is also shown as a MW standard (MW_HbA_ = 64 kDa), along with the hemoglobin of the bloodworm *Glycera dibranchiata* (GdHb), which includes a monomeric fraction (MW_GdHb monomer_ = 16 kDa)^57^ and a polymeric fraction (MW_GdHb polymer_ = 108 kDa).^58^ As expected for high MW proteins (e.g., MW_LtEc_ = 3.6 MDa), each Ec exhibited at least one high MW fraction (detected via absorbance at 280 nm) that quickly eluted from the column after 19 min. However, a second red fraction also eluted 4 min later for the EvEc, EhEc, and EeEc samples. The absorbance spectrum of the second red fraction observed with EvEc, EhEc, and EeEc was similar to most hemoglobins (data not shown), suggesting that globin subunits were present in the sample. The EeEc sample also displayed a minor third fraction with an elution time similar to HbA tetramer (28.5 min), but it was colorless. PAGE analysis revealed no change in band patterns between the SEC‐separated peaks and the original sample, indicating the presence of linker proteins. Therefore, it appears that EvEc, EeEc, and EhEc may have dissociated during tangential flow filtration (as was visibly observed during purification of AgEc) or it could reflect a structural instability of these Ecs at pH 7.4. Indeed, many other Ecs have been shown to dissociate at alkaline pH.^59^, ^60^, ^61^

### 2.4 Thermal stability

**Figure 3:**
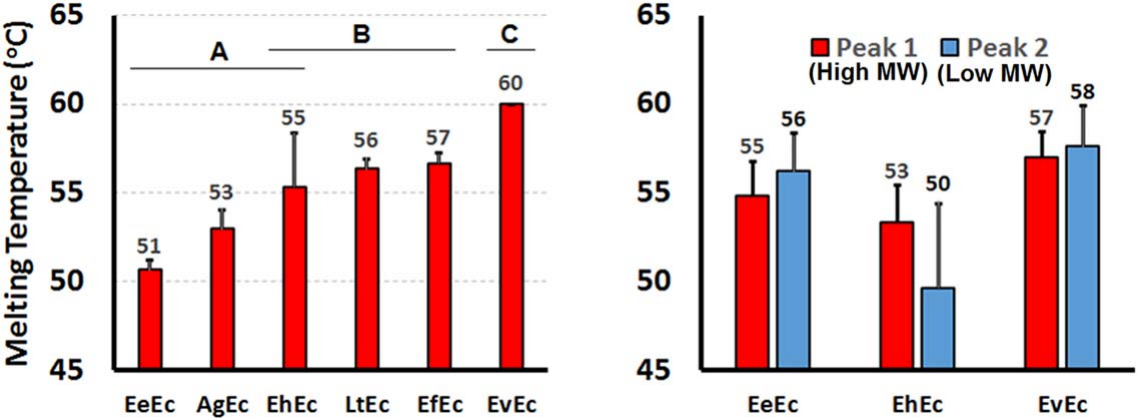
Melting temperatures of Ecs. Left: Thermal stability of Ec samples following TFF purification. Ascorbic acid (1 mg/ml) was added to these samples to ensure complete reduction, since heme oxidation is known to reduce thermal stability. Right: Comparison of the thermal stability of Ec fractions isolated by SEC. Peak 1 represents the high MW fraction that eluted first, while Peak 2 represents the lower MW fraction that eluted second

The thermal stability of the Ecs was compared by measuring their melting temperatures (*T*_m_) with a thermal shift assay (Figure 3). In this assay, SYPRO Orange dye binds to hydrophobic residues that are exposed as proteins denature at higher temperatures, causing an increase in dye fluorescence that can be quantitatively measured. The *T*_m_ is then defined as the point of inflection in this fluorescence plot, thereby indicating the temperature at which the proteins have started to denature. Overall, EvEc had the highest *T*_m_ (60°C), while EeEc (*T*_m_ = 51°C), and AgEc (*T*_m_ = 53°C) had the lowest melting temperatures. Meanwhile, EhEc, LtEc, and EfEc had a range of intermediate *T*_m_ values from 55 to 57°C. These values are similar to previously reported data for the Ec of the terrestrial worm *Glossoscolex paulistus* (52–54°C)^62^ and HbA (*T*_m_ = 55°C, data not shown).

Since EvEc, EhEc, and EeEc separated into two distinct fractions during SEC, the melting temperatures of those individual peaks were also compared (Figure 3, right). In each case, there were no significant differences in *T*_m_ between the two fractions of each Ec. However, it is interesting to note that both of the SEC‐purified EeEc fractions (55–56°C) were significantly more thermally stable than the TFF‐purified EeEc (51°C). These results suggest that a pro‐oxidant impurity (e.g., low MW protein or metal ion) may have been present in the TFF‐purified EeEc sample and then removed during SEC.

### 2.5 Oxygen affinity and cooperativity

**Figure 4:**
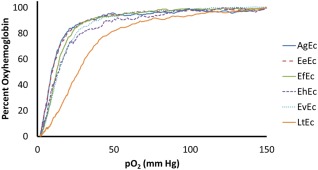
Representative oxygen equilibrium curves (OEC) of each Ec. All Ec samples were analyzed in Hemox Buffer at pH 7.4, supplemented with inert antifoam. All measurements performed at 37°C

Oxygen equilibrium curves for each Ec at 37°C in Hemox buffer are displayed in Figure 4, while their calculated oxygen affinity (*P*_50_) and cooperativity (i.e., Hill coefficient, *n*) values are shown in Table 2. As Figure 4 clearly shows, the Ecs can be separated into three categories based on O_2_ affinity. AgEc and EeEc have the highest O_2_ affinities (*P*_50_ = 9.29 and 9.68 mmHg, respectively), while the Ecs of the genera *Eisenia* have nearly identical mid‐range O_2_ affinities (*P*_50_ = 12.47–13.62 mmHg). In contrast, LtEc has a significantly lower O_2_ affinity (*P*_50_ = 26.25 ± 0.63 mmHg) than all of the other Ecs. Similar trends were also observed in Tris buffer and at 25°C (see Supporting Information Table 1). Interestingly, LtEc exhibits similar oxygen affinity to human whole blood (*P*_50_ = 26 mmHg^63^) while the oxygen affinities of the *Eisenia* Ecs are similar to pure human hemoglobin (*P*_50_ = 11 mmHg^25^).

**Table 2:** Oxygen affinity and cooperativity of Ecs at 37°C in hemox buffer pH 7.4

	LtEc	EvEc	EhEc	EfEc	EeEc	AgEc
Oxygen affinity (*P*_50_ mmHg)	26.25^a^ *± 0.63*	13.62^b^ *± 0.30*	13.01^bc^ *± 0.62*	12.47^c^ *± 0.29*	9.68^d^ *± 0.16*	9.29^d^ *± 0.15*
Hill coefficient (*n*)	2.39^a^ *± 0.02*	2.13^bc^ *± 0.01*	1.97^c^ *± 0.08*	2.39^a^ *± 0.09*	1.96^c^ *± 0.06*	2.16^b^ *± 0.11*

Superscripts indicate groups with significantly different (p < 0.05) values (a > b > c > d).

The Hill coefficients were similar among all of the Ecs (*n* = 2.13–2.39), except for the marginally lower values of *n* observed for EhEc and EeEc (1.97 ± 0.08 and 1.96 ± 0.06). Overall, LtEc has a significantly lower oxygen affinity than the other Ecs, while LtEc and EfEc have a significantly higher cooperativity.

### 2.6 Oxidation rate analysis

**Figure 5:**
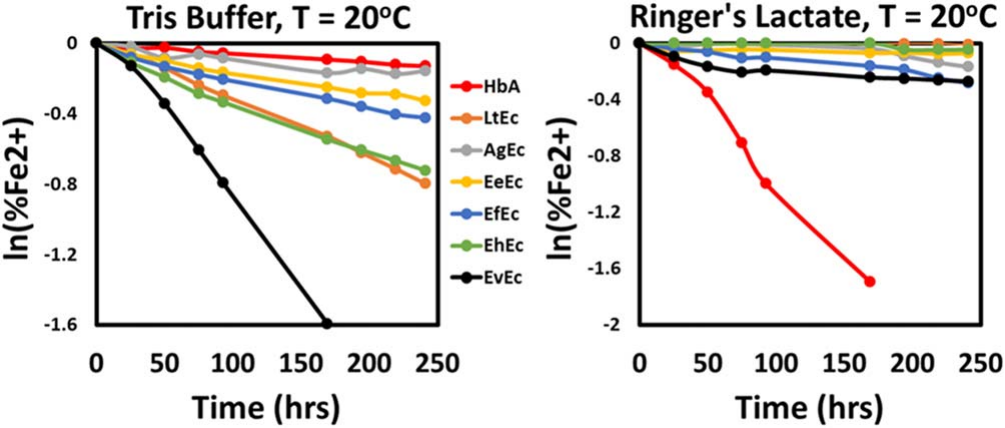
Representative first order oxidation rate plots. All Ec samples were initially normalized to an absorbance at 415 nm of *A* = 1.0 in 20 mM Tris Buffer or Ringer's Lactate solution. All measurements were performed at room temperature and pH 7.4

The oxidation of each Ec (Fe^2+^ to Fe^3+^) in Tris buffer (pH 7.4) and Ringer's Lactate solution (pH 7.4) at 25°C is shown in Figure 5, while the calculated oxidation rates (*k*_ox_) are shown in Table 3. In Tris buffer, all of the Ecs oxidized much faster than HbA (*k*_ox_ = 0.55 x 10^−3^ hr^−1^). Interestingly, the two highest oxidation rates were observed with EhEc and EvEc (3.4 x 10^−3^ and 9.8 x 10^−3^ hr^−1^, respectively), which were also observed to dissociate at pH 7.4 (see Figure 2). Other studies have shown a similar phenomenon, in which dissociation of Ecs significantly increases their oxidation rates.^64^, ^65^

**Table 3:** Oxidation rates Ecs after TFF purification

	HbA	AgEc	EeEc	EfEc	LtEc	EhEc	EvEc
Tris buffer (hr^−1^ x 10^3^)	0.55^a^ *± 0.04*	0.85^a^ *± 0.18*	1.41^a^ *± 0.18*	1.79^a^ *± 0.22*	3.17 *± 0.48*	3.35 *± 0.68*	9.79^a^ *± 1.65*
Ringer's lactate buffer (hr^−1^ x 10^3^)	11.34^a^ ^ǂ^ *± 2.07*	0.71^a^ *± 0.08*	0.25^a^ ^ǂ^ *± 0.09*	1.08^a^ ^ǂ^ *± 0.05*	0.04^a^ ^ǂ^ *± 0.05*	–	1.02^a^ ^ǂ^ *± 0.14*

a Asterisks (*) denote a statistically significant difference (p<0.05) between a sample and all other samples in that buffer, while double crosses indicate significant differences (p<0.05) between the Ec when it is in Tris and Ringer's lactate buffer.

In contrast, all of the trends observed in Tris buffer were reversed in Ringer's Lactate solution. The highest oxidation rate was observed with HbA (*k*_ox_ = 11.34 x 10^−3^ hr^−1^), while all of the Ecs oxidized at significantly lower rates. In fact, the oxidation rate of every Ec (except AgEc) was significantly lower in Ringer's Lactate compared to Tris buffer, while the oxidation of LtEc was also practically negligible (*k*_ox_ = 0.04 x 10^−3^ hr^−1^). This significant decrease in the oxidation rate of the Ecs in Tris buffer versus Ringer's Lactate solution may be due to the presence of two antioxidants, *N*‐acetyl l‐cysteine and sodium lactate, both of which have been shown to have antioxidant activity in vivo and in vitro, respectively.^66^, ^67^

## 3 DISCUSSION

These studies revealed several unique characteristics in each Ec that can be compared to determine the most promising candidate(s) for further development as a blood substitute. First, it is unlikely that AgEc is a viable blood substitute, since it oxidized quickly (%Fe^3+^ = 24.7%) and seemed to dissociate during purification. Some partial dissociation of EeEc, EhEc, and EvEc was also observed at pH 7.4, while LtEc, EfEc, and the remaining AgEc showed almost no dissociation. It is worth mentioning that LtEc has also been observed to dissociate above neutral pH, but at much higher pH values (>8.9).^68^ Additionally, studies with the Ec of the Brazilian earthworm *G. paulistus* have shown that oxidation of the heme iron can induce complete dissociation at pH values as low as 8.0 and the stability of GpEc is highest at acid pH (∼5.0).^60^, ^62^ Since our SEC experiments were conducted at a much lower pH of 7.4, it may be more likely that these Ecs dissociated due to shear stress during purification, though additional experiments need to be conducted to confirm this hypothesis. Nonetheless, these results strongly suggest that LtEc and EfEc may be especially promising blood substitutes, since they do not dissociate at physiological pH.

However, it is important to mention that *A. marina* Ec, which also dissociates at pH 7.4, has been reported to show promising results in preclinical animal trials.^30^ Therefore, the ability of each Ec to resist denaturation and oxidation during storage may be more important factors when selecting a blood substitute. The thermal shift assays in Figure 3 show that EvEc is the most thermally stable Ec (*T*_m_ = 60°C), albeit by a narrow margin (*T*_m_ = 57°C for EfEc and *T*_m_ = 56°C for LtEc). Unfortunately, EvEc (and EfEc) also exhibited relatively high oxidation rates in both Tris and Ringer's Lactate solutions. The other Ecs had relatively low oxidation rates in Ringer's Lactate solution (with antioxidants), but it is worth pointing out that the oxidation rate of LtEc (*k*_ox_ = 0.04 hr^−1^) was an order of magnitude lower than the other Ecs. Therefore, it may be possible to deploy LtEc for transfusion in remote areas or battlefield situations where a refrigerated blood bank facility (and therefore donated blood) is not available.

Finally, it is also interesting to note the significant difference in oxygen affinity between LtEc (*P*_50_ = 26.25 mmHg at 37°C) and the other Ecs (9.29–13.62 mmHg at 37°C). While the oxygen affinity of LtEc is approximately the same as that of human blood, it is currently unclear which level of oxygen affinity is desirable for an ideal blood substitute since direct in vivo comparisons of high and low oxygen affinity blood substitutes (or Ecs) have not yet been conducted. We attempted to determine the nature of the significant difference in O_2_ transport between LtEc and EfEc by aligning their amino acid sequences (Supporting Information Figure 2). Overall, most of the residues in each chain were highly conserved, especially within the heme pockets. Several mutations were detected in each chain, but most of them are unlikely to influence O_2_ affinity since they are oriented toward the solvent. However, a pattern of seven localized mutations (E30K, R33H, D34S, D37H, S47K, R118D, and H122D) does occur within the B and G helices of the D subunit. Three of these residues form inter and intrasubunit hydrogen bonds (D34) and salt bridges (R33, D37) in LtEc, but these bonds seem to be lost in EfEc. Disruption of these contacts could significantly influence O_2_ transport, since the B and G helices of the D subunit lie within the center of the A_3_B_3_C_3_D_3_ dodecamer (see Supporting Information Figure 2) and are, therefore, likely to be involved in the communication of allosteric changes between subunits. Indeed, similar mutations in the subunit interfaces of tetrameric hemoglobins have been shown to influence O_2_ affinity.^69^, ^70^

A few other key mutations between the LtEc and EfEc sequences were also observed that have been previously shown to influence the oxygen affinity of HbA. For example, the residue at position 116 in the D subunit of EfEc is a glutamine, while LtEc has a lysine at that position (N116K). The same mutation at the analogous position in the beta subunit of HbA (N108K) is known to decrease oxygen affinity.^71^ In addition, LtEc also has three other substitutions relative to EfEc (H103Q in the A subunit, along with Q132F and G145D in the D subunit) that have been shown to decrease the oxygen affinity of HbA (D94H, P124Q, and G136D, respectively).^72^, ^73^, ^74^ Overall, any or all of these isolated mutations could be responsible for the relatively low oxygen affinity of LtEc, but future mutational studies would be needed to confirm this hypothesis.

While our in vitro results suggest that oligochaete Ecs could be effective blood substitutes, their safety, O_2_ transport efficacy, and potential side effects still need to be determined in vivo. Preclinical studies in mice, rats, and hamsters have already shown that both LtEc and AmEc effectively deliver O_2_ in vivo without the significant increase in mean arterial pressure observed with some other HBOCs.^29^, ^30^, ^31^, ^75^ The circulation half‐life of both LtEc and AmEc are limited to 12 hours,^33^, ^75^ but PEGylation of LtEc has been shown to increase its half‐life up to 70 hours.^76^ Transfusions of LtEc and AmEc also do not elicit any changes in animal behavior or health for several months after the initial injection.^29^, ^31^ In addition, hyper‐responsive BP/2 mice injected with AmEc do not produce significant antibody titers against AmEc.^29^ All of these preliminary results are promising, but further work must be conducted to characterize the potential immunological effects of other Ecs and determine the potential need for other functions besides oxygen transport (e.g., carbon dioxide transport).^77^

## 4 MATERIALS AND METHODS

### 4.1 Preparation of human hemoglobin

Donated whole human blood was purchased from Interstate Blood Supply (Memphis, TN). Aliquots (50 ml) were centrifuged at 10,000 g for 5 min at 4°C and then the serum and white blood cell “buffy” layer was aspirated. The RBC pellet was then resuspended in 50 ml of 20 mM phosphate buffered saline (PBS, pH 7.4) This centrifugation/resuspension step was repeated three more times to ensure complete removal of the serum and white blood cells. After the final centrifugation step, the RBC pellet was resuspended in 50 ml of 20 mM Tris buffer (pH 7.4) and allowed to undergo hypotonic lysis overnight at 4°C (16–18 hr). The resulting lysate was centrifuged at 3,500 g for 15 min at 4°C to remove cell debris. The clarified crude HbA supernatant was decanted, then partially purified with ten successive rounds of diafiltration on a 10 kDa TFF filter as previously described.^78^

### 4.2 Preparation of crude Ecs

Approximately 500–1,000 worms of each species were purchased from various suppliers. *Lumbricus terrestris* specimens were purchased from Wholesale Bait Supply (Cincinnati, OH), while Uncle Jim's Worm Farm (Spring Grove, PA) provided *Eisenia fetida*, Knutson's Live Bait (Brooklyn, MI) provided *Eisenia veneta*, and Worms4Earth (Pensacola, FL) provided *Eisenia hortensis*, *Eudrilus eugeniae,* and *Amynthas gracilis*. All worms were purified in a similar manner, as shown in Supporting Information Figure 3. Worms were initially rinsed with tap water to remove dirt and then briefly homogenized in a blender for 10 s. The resulting homogenate was immediately centrifuged at 3,500 g for 30 min at 4°C to remove solid impurities. The cloudy red supernatant was decanted and centrifuged again at 15,000 g for 30 min at 4°C. The clarified Ecs were then sterilized by passing the crude hemoglobin solution through a TFF cartridge with 0.2 µm pore size with a surface area of 790 cm^2^ (Spectrum Labs, Rancho Dominguez, CA). Large quantities of small molecular weight protein impurities were removed from each Ec sample by 10 successive rounds of diafiltration using a 500 kDa TFF filter as previously described.^33^ Specifically, in each round of diafiltration, the red retentate was concentrated 10‐fold and then diluted again (e.g., −500 to 50 ml) with 20 mM Tris buffer, pH 7.0 at 4°C. After the final concentration step, the cyanmethemoglobin assay^79^ was performed to measure oxidation levels and the samples were frozen at −72°C until needed.

### 4.3 Cyanmethemoglobin oxidation assay

Each oxidation assay was performed using clear 96 well plates and a Synergy HT Microplate Reader (BioTek, Winooski, VT) using the cyanmethemoglobin method described previously.^79^ For each measurement, the hemoglobin sample was diluted with 20 mM Tris buffer, pH 7.0 by a factor, *D*_1_, to a total volume of 150 μl until the absorbance at 630 nm (*A*_630_) was in the range of 0.1–1.0. The initial *A*_630_ reading was recorded (*A*_1_), 20 μL of 10% KCN was added to the sample well, and the new *A*_630_ reading was recorded again (*A*_2_). The concentration of oxidized hemoglobin [Hb : Fe^3+^] for each sample was then calculated using the following equation (*λ*_1_ = 0.45 cm, *λ*_2_ = 0.51 cm, *ɛ*_1_ = 3.7 cm^−1^ mM heme^−1^):

(1) $$[heme:Fe^{3+}]=\left[\left(\frac{A_{1}}{\lambda_{1}}\right)-\left(\frac{A_{2}}{\lambda_{2}}\right)\right]\left(\frac{D_{1}}{{ɛ}_{1}}\right)$$

For each Ec, a separate sample was diluted by a factor, *D*_2_, to a total volume of 150 μl until the absorbance at 540 nm (*A*_540_) was in the range of 0.3–1.0. Then, 20 μl of 10% potassium ferricyanide K_3_[Fe(CN)_6_] was added to each sample and incubated at room temperature for 2 min. Finally, 20 μl of 10% KCN was added to the sample and the *A*_540_ was recorded as *A*_3_. The total concentration of hemoglobin [Hb_total_] was calculated using the following equation (*λ*_3_ = 0.57 cm, *ɛ*_2_ = 11.0 cm^−1^ mM heme^−1^):

(2) $${[heme}_{total}]=A_{3}*\left(\frac{D_{2}}{\lambda_{3}{ɛ}_{2}}\right)$$

The percent oxidation of each hemoglobin sample, shown in Table 1, was calculated by dividing oxidized hemoglobin concentration, [Hb : Fe^3+^], by total hemoglobin, [Hb_total_]. Each purification yielded ∼50 ml of ∼20–85 µM heme per 100 worms (data not shown).

### 4.4 SEC analysis

Each Ec and Hb was analyzed using a NGC^TM^ Chromatography System (BioRad, Hercules, CA) with a BioRad Enrich^TM^ SEC 650 10 x 300 column with a total volume of 24 ml (Cat. #780–1650). Samples were eluted from the column using 20 mM Tris buffer, pH 7.4 at a flow rate of 0.5 ml/min. Distinct peak fractions were detected via absorbance at 280 nm and collected separately for further analysis (e.g., thermal stability and PAGE). The elution profiles shown in Figure 2 were normalized such that the maxima of the first elution peak were held constant.

### 4.5 PAGE analysis

Twelve percent polyacrylamide resolving gels were prepared by mixing the following reagents: Millipore water (1.7 ml), 1.25 ml resolving gel buffer (1.5 M Tris‐HCl, pH 8.8), 2.0 ml 30% acrylamide, 50 µl 10% SDS, 25 µl 10% ammonia persulfate, and 2.5 µl TEMED (tetramethylethylenediamine). Stacking gels were made by mixing the following reagents: 1.4 ml Millipore water, 0.39 ml stacking gel buffer (0.5 M Tris‐HCl, pH 6.8), 0.75 ml 30% acrylamide, 30 µl 10% SDS, 15 µl 10% ammonia persulfate, and 3.0 µl TEMED. The gels were run with a 0.05 M Tris, 0.38 M glycine, 0.2% SDS, pH 9.0 running buffer. All Ec and HbA samples were diluted to a final absorbance of *A*_540 nm_ = 0.1 (∼7 µM heme) in 20 mM Tris buffer, pH 7.0 and mixed in a 1:1 volume ratio with a Laemmli buffer containing β‐mercaptoethanol and incubated at 95°C for 10 min. Each gel was initially run at 30 volts for 10 min to separate excess salts, then the voltage was increased to 125 volts for approximately 2 hr. The gels were then stained overnight in staining solution (0.25% Brilliant Blue R (Sigma Aldrich, B0149), 10% acetic acid, 45% ethanol, 45% water). The gels were then destained in 2–3 rounds of ∼200 ml destaining solution (10% acetic acid, 20% ethanol, 70% water).

### 4.6 SYPRO orange thermal stability assay

SYPRO Orange Dye (ThermoFisher, Cat. # S6650) was diluted with DMSO from a 5,000x stock to a final concentration of 200x, while the Ecs were diluted to a final absorbance of *A*_525_ = 0.0216 and HbA was diluted to a final absorbance of *A*_520_ = 0.2 in 10 mM HEPES buffer. Ascorbic acid was also added to each diluted sample at a final concentration of 1 mg/ml to ensure that the iron in the heme groups was fully reduced (reduction of the heme is necessary, since it has been shown that oxidation of the heme iron reduces thermal stability in a similar terrestrial Ec, GpEc^80^). Forty‐five microliters of each Ec sample was added to a MicroAmp Optical 96‐Well Reaction Plate (Applied Biosystems, ref# 4306737) and mixed with 5 uL of the 200x SYPRO Orange dye and analyzed on a 7300 Real‐Time PCR System (Applied Biosystems, Foster City, CA). Specifically, the fluorescence of each well was monitored while the temperature was increased in 1°C increments from 30 to 89°C. The raw fluorescence data were then analyzed to detect points of inflection which indicate the protein's melting temperature (*T*_m_).

### 4.7 Oxygen affinity (*P*_50_) and the hill coefficient (*n*)

Oxygen equilibrium curves were generated using a Hemox Analyzer (TCS Scientific, New Hope, PA). For each run, the sample was diluted in Hemox buffer (135 mM NaCl, 5 mM KCl, 30 mM TES, pH 7.4 with antifoam) and sparged with air until the partial pressure of O_2_ (pO_2_) reached ∼150 mmHg of O_2_. The sample was then sparged with pure N_2_ until the pO_2_ decreased to ∼2 mmHg while the absorbance of the sample was monitored to determine the percent O_2_ saturation of the hemoglobin. *P*_50_ values were defined as the pO_2_ at which half of the hemoglobin binding sites were bound to O_2_. Hill coefficients (*n*) were calculated with Equation (3):

(3) $$\log\left(\frac{{HbO}_{2}}{1-{HbO}_{2}}\right)=n\log\left(\frac{{pO}_{2}}{P_{50}}\right)$$

where *n* is the Hill coefficient, pO_2_ is the unbound oxygen concentration and HbO_2_ is the fraction of occupied O_2_ binding sites. In this context, Hill coefficients greater than one represent positive cooperativity and allosteric interactions between subunits, while *n* = 1 suggests noncooperative O_2_ binding.

### 4.8 Oxidation rate analysis

Ecs were initially diluted in 20 mM Tris or Ringer's Lactate buffers (pH 7.0) to *A*_415_ = 1.0 (l = 0.3 cm) and then sterilized with a 0.2 µm sterile syringe filter in a biological safety cabinet. The solutions were then separated into 200 µl aliquots and stored in the dark at room temperature. The absorbance spectra of the samples were measured daily for 2 weeks and then used to estimate oxidation levels using Equation (4). Since the plots of ln(%Fe^2+^) versus time for each Ec appeared to be linear, a single exponential decay model (Equation (5)) was used to estimate oxidation rates for each Ec.

(4) $$\%{Fe}^{2+}=\frac{{\left(\frac{A_{415}}{A_{405}}\right)}_{t}-{\left(\frac{A_{415}}{A_{405}}\right)}_{100\%\;Fe3+}}{{\left(\frac{A_{415}}{A_{405}}\right)}_{100\%\;Fe2+}-{\left(\frac{A_{415}}{A_{405}}\right)}_{100\%\;Fe3+}}$$

(5) $$ln\left(\%{Fe}^{2+}\right)={-k}_{ox}t$$

### 4.9 Sequence alignment

The amino acid sequences of the LtEc globin chains were obtained from UniProt (Accession #s: *A* = P13579.1, *B* = P02218.2, *C* = P11069.3, *D*_1_ = U55073, *D*_2_ = U55074). The amino acid sequence of the EfEc globin chains was retrieved from the expressed sequence tag (EST) library using Accession #s HO001180.1, GO269560.1, EH671066.1, HO001517.1, and EH670311.1. The multiple sequences were then aligned using ClustalW2 and the 3D structure of LtEc (PDB ID 2GTL) was visualized using SwissPDB Viewer.

### 4.10 Statistical analysis

All statistical analyses were performed using R Studio software (Boston, MA) or by simultaneous *T* tests in Microsoft Excel. Statistical significance was defined as *p* < .05. All analyses conducted in R Studio were parametric analysis of variance and Tukey's Honestly Significant Difference following tests of normality and homogeneity of variances.

## 5 CONCLUSIONS

Altogether, these results identify LtEc and EfEc as two promising potential blood substitutes that warrant future study. They both resist oligomeric dissociation at pH 7.4, while also exhibiting relatively low oxidation rates and high melting temperatures. It is also interesting to note that these Ecs have vastly different oxygen affinities, but the implications of that difference will need to be determined in future animal studies. Finally, it would also be interesting to directly compare the terrestrial Ecs studied in this work to other well‐studied marine Ecs (e.g., AmEc).

## Supporting information

Additional Supporting Information may be found online in the supporting information tab for this article.

Supporting Figures
