# Supplementary material for: Direct comparison of oligochaete erythrocruorins as potential blood substitutes
Source: Bioeng Transl Med. 2017 Jul 19;2(2):212–21. doi: 10.1002/btm2.10067 (PMC5675092; doi:10.1002/btm2.10067)
Supplement: Supplementary file 1 — Supporting Figures [file BTM2-2-212-s001.docx]

Supplementary Information for:

Direct Comparison of Oligochaete Erythrocruorins as Potential Blood Substitutes

**Devon Zimmerman^a^, Matt DiIusto^a^, Jack Dienes^a^, Osheiza Abdulmalik^b^, Jacob J. Elmer^a^**

a – Villanova University, 800 East Lancaster Avenue, Villanova, PA 19085

b – Division of Hematology, Abramson Building, The Children's Hospital of Philadelphia, 34th St. & Civic Center Blvd, Philadelphia, PA 19104

Corresponding author: Jacob J. Elmer, Department of Chemical Engineering, Villanova University, 217 White Hall, 800 East Lancaster Avenue, Villanova, PA 19085, Telephone: (610) 519-3093, E-mail: Jacob.Elmer@Villanova.edu


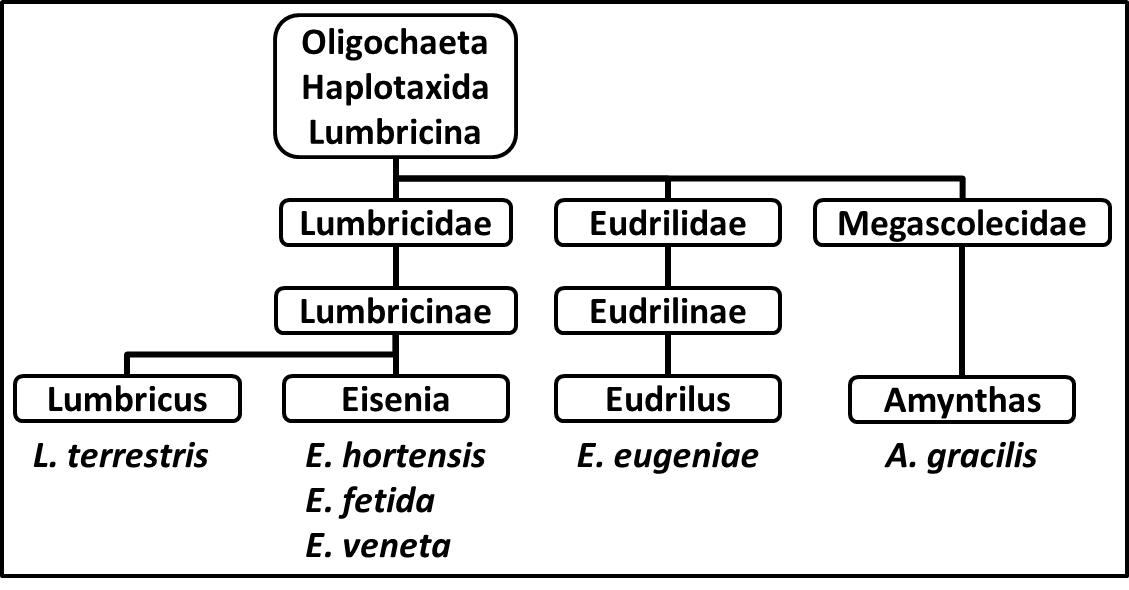


**SI Figure 1**: Phylogeny of terrestrial worm species used in this study. All species are oligochaetes, but are separated into four different genera: *Lumbricus*, *Eisenia*, *Eudrilus*, and *Amynthas.*

*
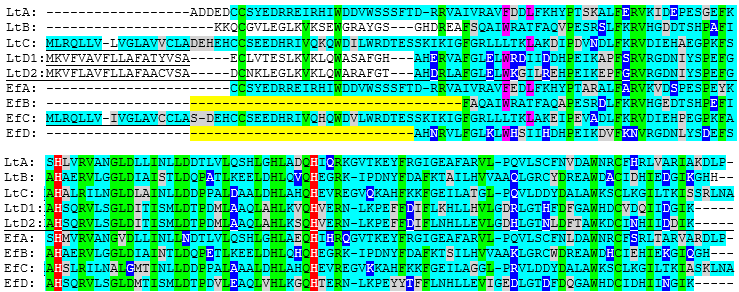
*

*
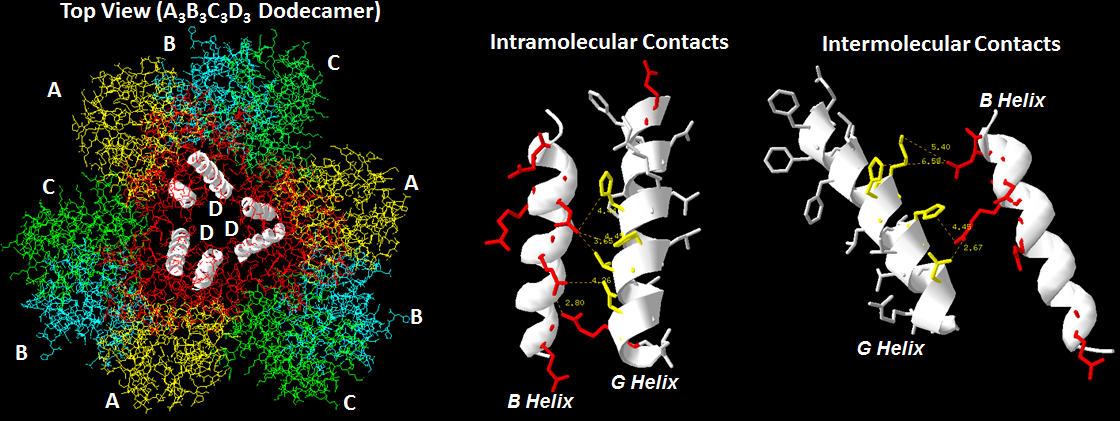
*

**SI Figure 2:** Top: Sequence alignment of LtEc and EfEc globin chains. The canonical distal and proximal histidines are shown in red, while other highly conserved residues occurring in all chains are highlighted in green. Residues conserved between analogous globin subunits (LtA and EfA) are highlighted in light blue with dark text. Significant mutations are highlighted in dark blue with white text. Note: the A helix of the B and D globins for EfEc were unavailable (highlighted in yellow). Bottom: Significantly mutated residues (highlighted in red) occurring between LtEc and EfEc in the B and G helices of the D globin subunit.

**SI Table 1**: Total Oxygen Affinity and Cooperativity of Ecs in Tris and Hemox Buffers

|  | **T** |  | **LtEc** | **EeEc** | **AgEc** | **EhEc** | **EvEc** | **EfEc** |
| --- | --- | --- | --- | --- | --- | --- | --- | --- |
| **Oxygen Affinity**  **(P_50_  mm Hg)** | 25^o^C  Tris |  | 17.57  *± 0.91* | 7.45  *± 1.00* | 7.43  *± 0.66* | 6.89  *± 0.63* | 6.82  *± 0.59* | 6.59  *± 0.17* |
|  | 37^o^C  Tris |  | 37.61  *± 2.74* | 12.42  *± 0.57* | 14.29  *± 1.04* | 13.53  *± 0.27* | 13.33  *± 0.14* | 12.42  *± 0.49* |
|  | 37^o^C  Hemox |  | 26.25  *± 0.63* | 9.68  *± 0.16* | 9.29  *± 0.15* | 13.01  *± 0.62* | 13.62  *± 0.30* | 12.47  *± 0.29* |
| **Hill Coefficient**  **(n)** | 25^o^C  Tris |  | 1.83  *± 0.01* | 1.80  *± 0.17* | 1.38  *± 0.27* | 1.79  *± 0.27* | 1.98  *± 0.23* | 1.64  *± 0.07* |
|  | 37^o^C  Tris |  | 1.66  *± 0.05* | 1.93  *± 0.04* | 1.66  *± 0.08* | 1.89  *± 0.02* | 1.99  *± 0.17* | 1.59  *± 0.06* |
|  | 37^o^C  Hemox |  | 2.39  *± 0.02* | 1.96  *± 0.06* | 2.16  *± 0.11* | 1.97  *± 0.08* | 2.13 *± 0.01* | 2.39  *± 0.09* |


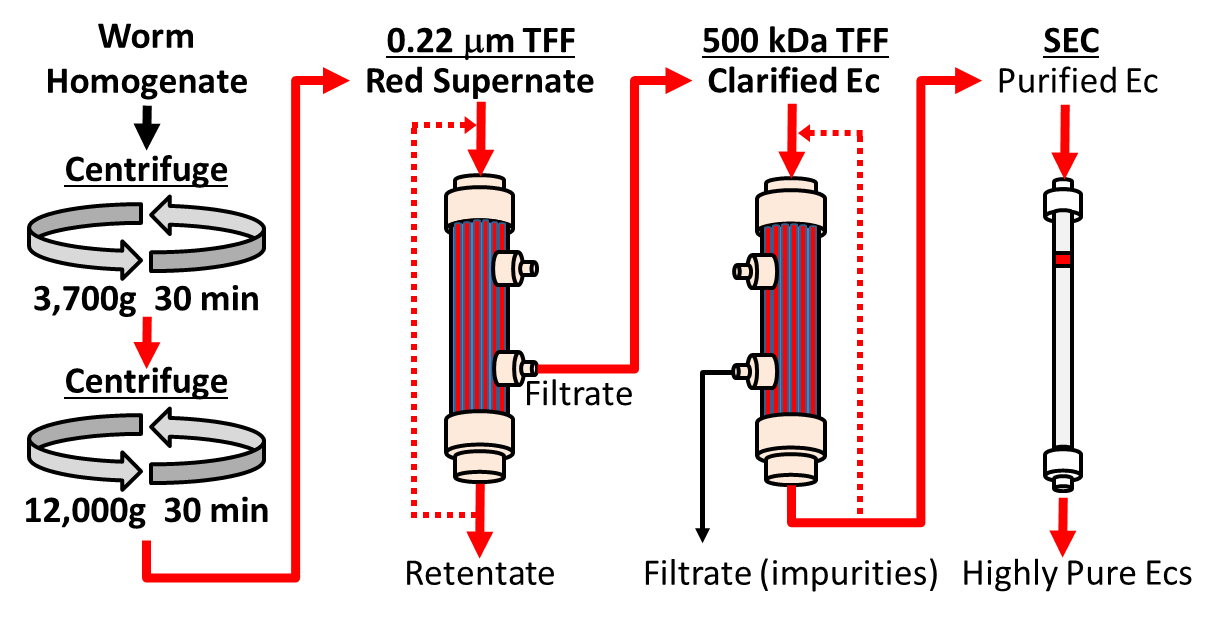


**SI Figure 3**: Purification scheme. All Ecs were purified via homogenization of the corresponding worms, followed by two rounds of centrifugation (3,500 g for 30 min, then 15,000 g for 30 min). The red supernatant was then clarified with a 0.2 um TFF filter and Ecs were purified with a 500 kDa MWCO TFF filter. In some experiments, samples were then purified further using a size exclusion column with a size separation range of 5-650 kDa.
